# Kinetic Processes in Amorphous Materials Revealed by Thermal Analysis: Application to Glassy Selenium

**DOI:** 10.3390/molecules24152725

**Published:** 2019-07-26

**Authors:** Jiří Málek, Roman Svoboda

**Affiliations:** Department of Physical Chemistry, Faculty of Chemical Technology, University of Pardubice, Studentská 95, 53210 Pardubice, Czech Republic

**Keywords:** glass, structural relaxation, crystallization, viscosity, thermal analysis

## Abstract

It is expected that viscous flow is affecting the kinetic processes in a supercooled liquid, such as the structural relaxation and the crystallization kinetics. These processes significantly influence the behavior of glass being prepared by quenching. In this paper, the activation energy of viscous flow is discussed with respect to the activation energy of crystal growth and the structural relaxation of glassy selenium. Differential scanning calorimetry (DSC), thermomechanical analysis (TMA) and hot-stage infrared microscopy were used. It is shown that the activation energy of structural relaxation corresponds to that of the viscous flow at the lowest value of the glass transition temperature obtained within the commonly achievable time scale. The temperature-dependent activation energy of crystal growth, data obtained by isothermal and non-isothermal DSC and TMA experiments, as well as direct microscopic measurements, follows nearly the same dependence as the activation energy of viscous flow, taking into account viscosity and crystal growth rate decoupling due to the departure from Stokes–Einstein behavior.

## 1. Introduction

**Figure 1:**
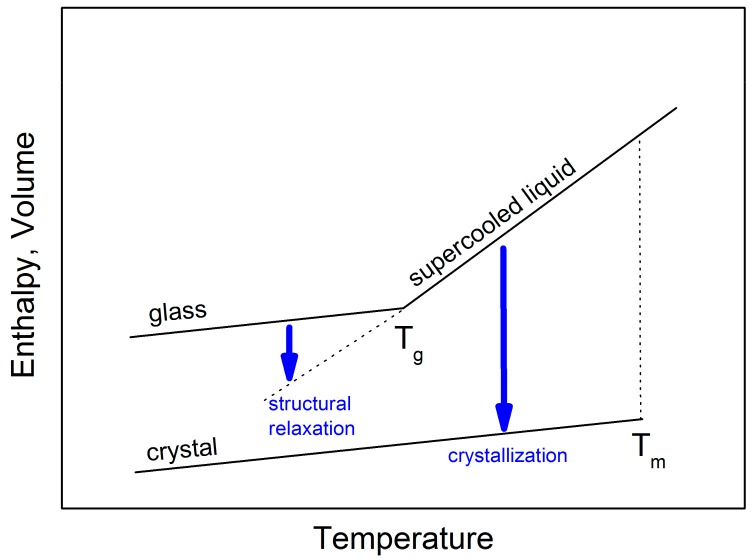
Schematic diagram showing the change in enthalpy and volume during glass formation, structural relaxation and crystallization.

Glasses are amorphous materials lacking the periodic atomic arrangement typical for crystalline substances. Structurally, they resemble supercooled liquids but behave mechanically like solids. Figure 1 shows the specific volume or enthalpy as a function of temperature for a typical glass-forming liquid. Upon slow cooling from high temperatures, a liquid may crystallize at T_m_ forming a stable crystalline material. However, if the cooling through this temperature range is fast enough to avoid nucleation and subsequent crystal growth, a metastable supercooled liquid state is attained. 

When a supercooled liquid is cooled by a cooling rate of q^+^_1_ to lower temperatures, the internal molecular motion slows down and its viscosity significantly increases. At the glass transition temperature (T_g_), the time needed for molecular rearrangement becomes comparable to the experimental time scale. At a lower cooling rate (q^+^_2_ < q^+^_1_) the supercooled liquid stays in metastable equilibrium until lower temperatures. Therefore, the glass transition is not a true thermodynamic phase transition depending on procedural variables such as cooling rate. As a consequence, there is not a single glassy state, and the properties of the glass depend upon how it was obtained [1].

Clear evidence of the non-existence of a single glassy state is a slow, gradual approach of volume or enthalpy towards the extrapolated supercooled liquid equilibrium line that has been called “structural relaxation”. This process, associated with a slow molecular rearrangement, is experimentally observable in the glass transition range (see Figure 1). It seems that structural relaxation is strongly affected by the supercooled liquid dynamics. An understanding of clues between long time-scale structural relaxation and short time-scale molecular dynamics of corresponding supercooled liquids is of fundamental importance for glass science.

On reheating of a glass, the structural relaxation peak is usually observed just above T_g_. At higher temperatures the crystallization process takes place [2]. These processes can be followed by thermal analysis methods such as differential thermal analysis (DTA) or differential scanning calorimetry (DSC). Both these methods are quite frequently used to study the structural relaxation and crystallization behavior in glasses. Usually, the kinetic parameters of such activation energies are extracted from experiments taken at different heating rates. In this paper, the physical meaning of these parameters determined for structural relaxation and crystallization of glassy selenium is analyzed and discussed with respect to the viscosity behavior of supercooled selenium.

## 2. Results

This section provides a concise description of the structural relaxation experiments as well as the crystallization of glassy selenium obtained by differential scanning calorimetry (DSC) and dilatometry.

### 2.1. Volume and Enthalpy Relaxation

The isothermal volume relaxation is typically studied by a temperature down-jump experiment. In this experimental set-up the classical mercury-filled dilatometer containing a selenium glass is first equilibrated in a thermal bath at temperature T_0_. Then it is quickly transferred to another temperature bath at temperature T < T_0_ and the time-dependent volume contraction is recorded. The temperature up-jump experiment starts by equilibration of a dilatometer at a temperature T_0_. Then it is quickly transferred to another thermal bath at temperature T > T_0_ and the time dependent volume expansion is recorded. Isothermal volume relaxation can be expressed as a relative departure of an actual volume V from the equilibrium volume V_∞_:

(1) $$\delta =\frac{V-V_{\infty }}{V_{\infty }}.$$

**Figure 2:**
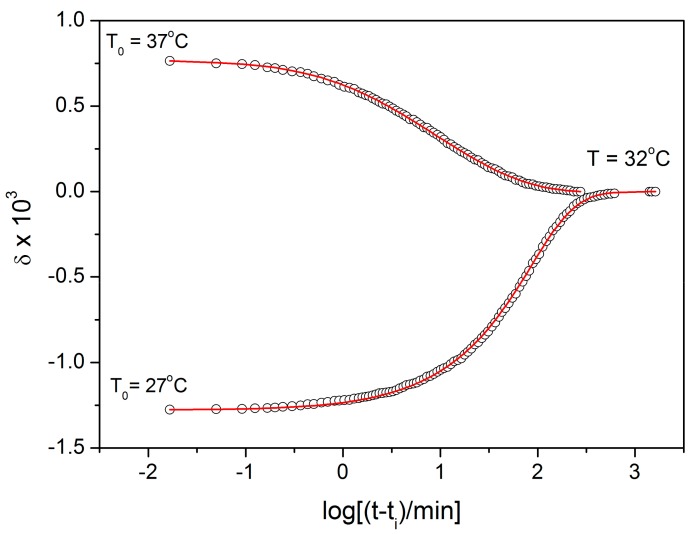
The volume change of Se glass subjected to a temperature down-jump and up-jump ±5 °C. Points correspond to experimental data obtained by dilatometry. Full lines were calculated for the Tool–Narayanaswamy–Moynihan (TNM) model (parameters in text).

The relaxation response immediately after the temperature jump from temperature T_0_ to T is defined by the following equation:

(2) $$\delta_{0}=\Delta \alpha \left(T_{0}-T\right),$$

where Δα corresponds to the difference between the thermal expansion coefficient in a selenium supercooled liquid and selenium glass [3]. Figure 2 shows the isothermal volume relaxation response of selenium glass subjected to the temperature down-jump (T_0_ = 37 °C, T = 32 °C) and up-jump (T_0_ = 27 °C, T_B_ = 32 °C). Open circles represent experimental data. The logarithmic time axis is normalized to the initial time that corresponds to the thermal equilibration of dilatometer after the temperature jump (t_i_ = 70 s) [3].

It is clearly seen that both relaxation responses after temperature down-jump and up-jump are non-exponential and non-linear. Such behavior can be described by a non-exponential decay function including the reduced time integral [4]:

(3) $$\delta \left(t\right)=\delta_{0}\exp\left[-{\left(\int_{0}^{t}\frac{dt}{\tau (T,T_{f})}\right)}^{\beta }\right],$$

where β is the non-exponentiality parameter (0 < β ≤ 1), inversely proportional to the width of the spectrum of relaxation times. It is assumed that τ depends on temperature T, as well as on the instantaneous structure of amorphous material characterized by the fictive temperature T_f_ [5]. The relaxation time can be expressed by the following equation [6]:

(4) $$\tau (T,T_{f})=A_{rel}\cdot \exp\left[x\frac{E_{rel}}{RT}+(1-x)\frac{E_{rel}}{RT_{f}}\right],$$

where A_rel_ is the pre-exponential constant, x is the non-linearity parameter (0 < x ≤ 1) and E_rel_ is the effective activation energy of the relaxation process. The time-dependent fictive temperature for this Tool–Narayanaswamy–Moynihan (TNM) model can be expressed as

(5) $$T_{f}=\frac{\delta (t)}{\delta_{0}}\left[T_{0}-T\right]+T.$$

Full lines in Figure 2 were calculated by using Equations (3)–(5) for the following set of parameters: β = 0.58 ± 0.05, x = 0.42 ± 0.05, ln (A_rel_/s) = −133.0 ± 0.5 and E_rel_ = 355 ± 2 kJ·mol^−1^ [3]. 

Enthalpy relaxation cannot be measured directly in a similar way. However, the DSC heating scans exhibit a typical relaxation overshot just above the glass-transition temperature. It can be shown that normalized heat capacity (C_p_^N^) measured by DSC can be expressed [6] as the first derivative of the fictive temperature dT_f_/dT:

(6) $$C_{p}^{N}=\frac{C_{p}-C_{pg}}{C_{pl}-C_{pg}}=\frac{dT_{f}}{dT},$$

where C_p_ is measured heat capacity, C_pg_ is heat capacity of a glass and C_pl_ is heat capacity of a supercooled liquid.

**Figure 3:**
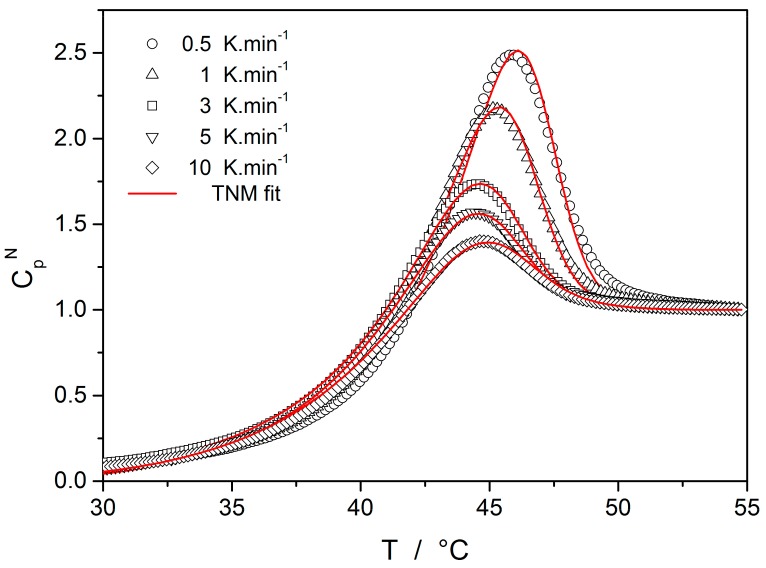
The normalized heat capacity of Se glass reflecting the structural relaxation in the glass transition range. Points correspond to selected experimental data-curves obtained by differential scanning calorimetry (DSC). Full lines were calculated for the TNM model (parameters in text).

The temperature dependent plots of C_p_^N^ are shown in Figure 3. Open circles represent experimental data for heating scans at q^+^ = 10 K·min^−1^ taken immediately after the cooling scans performed at the indicated rates. Full lines in Figure 3 were calculated by using Equations (3)–(6) for the following set of parameters: β = 0.65 ± 0.05, x = 0.52 ± 0.05, ln (A_rel_/s) = −133.0 ± 0.5 and E_rel_ = 355 ± 2 kJ·mol^−1^ [3]. The parameters ln (A_rel_/s) and E_rel_ are identical as for the volume relaxation. However, the values of non-exponentiality and non-linearity parameters are higher than those obtained for volume relaxation.

Very similar results are also obtained for the thermal expansion coefficient measured by thermomechanical analysis for As_2_Se_3_ glass [7,8]. The agreement between the experiment and the TNM model is very good below T_g_. Nevertheless, some deviations are observed in the supercooled liquid above T_g_ due to viscous flow deformation of the sample. Such effect is not relevant in classical mercury dilatometry or DSC experiments described above.

### 2.2. Nucleation and Crystal Growth

On further reheating of the supercooled liquid above T_g_, the nucleation process takes place being followed by crystal growth. In a selenium supercooled liquid, crystals grow from a relatively low-density nuclei population. Well defined and compact spherulitic structures grow from these centers. It seems that the nucleation has negligible effects during the crystal growth. The crystal growth is usually visible on a microscopic level and therefore it can directly be observed by microscopy methods using hot stage.

**Figure 4:**
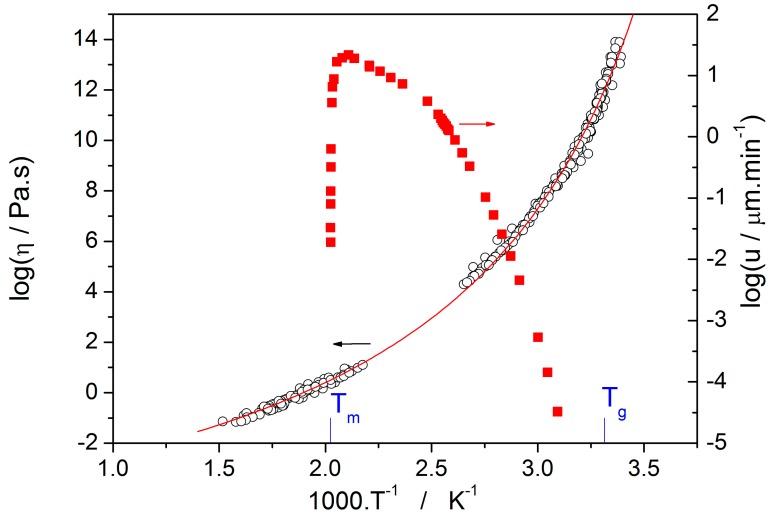
The crystal growth rate of isothermally grown spherulitic crystals and viscosity of a selenium supercooled liquid as a function of reciprocal temperature.

Temperature-dependent data for crystal growth velocity u [9,10] and viscosity η [10,11,12] in a selenium supercooled liquid are shown in Figure 4. These two kinetic processes are closely bound together as will be discussed later.

We can assume that in a narrow temperature range the crystal growth velocity can be described by a simple exponential dependence on reciprocal temperature u ~ exp(−E_G_/RT), that should be linear on a logarithmic scale. The activation energy of crystal growth E_G_ can then be obtained from the slope of such linear dependence,

(7) $$\frac{d\log\left(u\right)}{d\left(1/T\right)}=-\frac{E_{G}}{2.303\cdot R},$$

where the coefficient 2.303 in Equation (7) comes from the conversion from the natural to the decimal logarithm.

From the crystal growth rate data shown in Figure 4 it is clearly seen that the activation energy is gradually decreasing from a relatively high value just above T_g_ (≅250 kJ·mol^−1^) to a considerably lower value (≅40 kJ·mol^−1^) just below the maximum growth rate.

**Figure 5:**
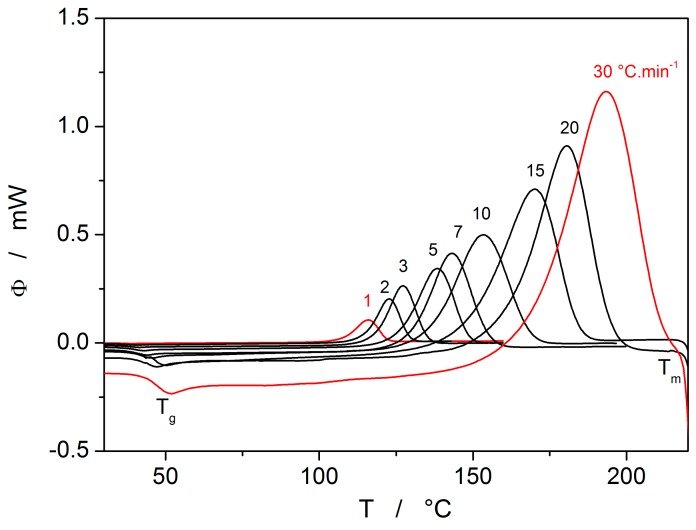
The DSC measurement of bulk selenium glass at different heating rates.

The heat evolved during the crystal growth can easily be recorded by DSC. Figure 5 shows such heat flow at different scanning rates ranging from 1 to 30 K·min^−1^.

The crystallization peaks shown in Figure 5 involve the whole crystallization process including nucleation and crystal growth. More detailed calorimetric studies [13,14,15,16] indicate a complex behavior involving both bulk and surface crystal growth with nucleation possibly affected by internal stresses. Nevertheless, nucleation and surface crystal growth are negligible for the bulk selenium sample.

The DSC curves can easily be converted to kinetic data. It is assumed that the fraction crystallized, α, can be obtained by partial integration of non-isothermal heat flow, ϕ, after baseline subtraction:

(8) $$\alpha =\frac{1}{\Delta Hq^{+}}\int_{T_{on}}^{T}\phi \cdot dT,$$

where ΔH_c_ correspond to enthalpic change of crystallization, q^+^ is the heating rate and T_on_ is the starting point of baseline approximation. The heat flow due to the crystallization process can then be written as

(9) $$\phi =\Delta H_{c}A\cdot \exp\left(-E_{c}/RT\right)\cdot f\left(\alpha \right),$$

where A is the preexponential factor and E_c_ is the activation energy of the crystallization process. The f(α) function corresponds to the Johnson–Mehl–Avrami–Kolmogorov (commonly abbreviated as JMA) model of nucleation-growth process:

(10) $$f\left(\alpha \right)=m\left(1-\alpha \right){\left[-\ln\left(1-\alpha \right)\right]}^{1-1/m}.$$

The development of this equation is described in [8] and references quoted in.

Analysis of DSC curves shown in Figure 5 is complicated due to the strong temperature dependence of heat flow as follows from Equation (9). Nevertheless, it has been shown [17,18] that if the measured heat flow is multiplied by T^2^ and plotted as a function of α, all data taken at different heating rates collapse to one master curve defined as

(11) $$z\left(\alpha \right)=f\left(\alpha \right)\int_{0}^{\alpha }\frac{d\alpha }{f\left(\alpha \right)}≅\phi \cdot T^{2}.$$

This function can be expressed for the JMA model as follows:

(12) $$z\left(\alpha \right)=m\left(1-\alpha \right)\left[-\ln\left(1-\alpha \right)\right].$$

**Figure 6:**
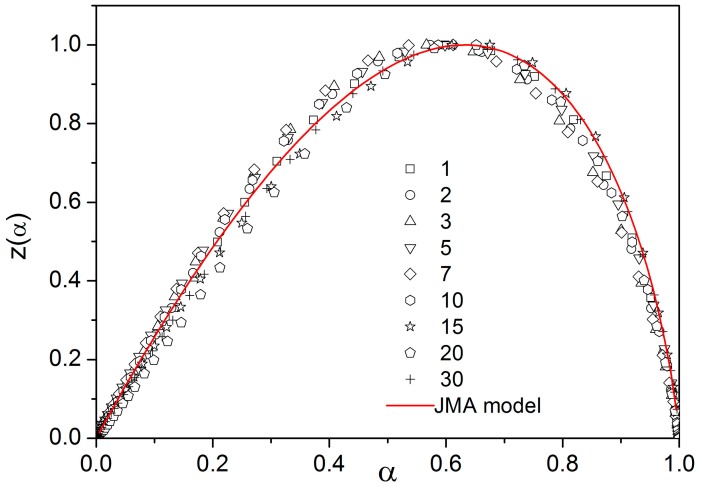
The z(α) function obtained by transformation of DSC data shown in Figure 5. Points were calculated by using Equation (11) (numbers indicate heating rate). The full line was calculated by Equation (12).

Figure 6 shows the z(α) function obtained by transformation of all crystallization peaks from Figure 5 by Equations (8) and (11). These plots are scaled within the 0 < z(α) ≤ 1 range for easier comparison of different data sets (points). The z(α) function (full line) calculated by Equation (12) fits the experimental data quite well. This confirms the applicability of the JMA model for the description of non-isothermal crystallization kinetics in selenium glass. Another method to test the validity of this model is the shape analysis of DSC curve [19]. Slight data scatter might indicate variation in thermal contacts between the sample and DSC sensor.

The activation energy of the non-isothermal crystallization process E_c_ can be determined by the Kissinger method [20] from the shift of the maximum of the DSC peak T_p_ with heating rate q^+^:

(13) $$\frac{d\ln\left(q^{+}/T_{p}^{2}\right)}{d\left(1/T_{p}\right)}=-\frac{E_{c}}{R}.$$

In case of the isothermal data, the Friedman method [21] is usually used, where (d*α*/d*t*)*_α_* and *T_α_* are the conversion rate and temperature corresponding to arbitrarily chosen values of conversion *α*:

(14) $$\ln\left({\left[d\alpha /dt\right]}_{\alpha }\right)=-\frac{E}{RT_{\alpha }}+const..$$

**Figure 7:**
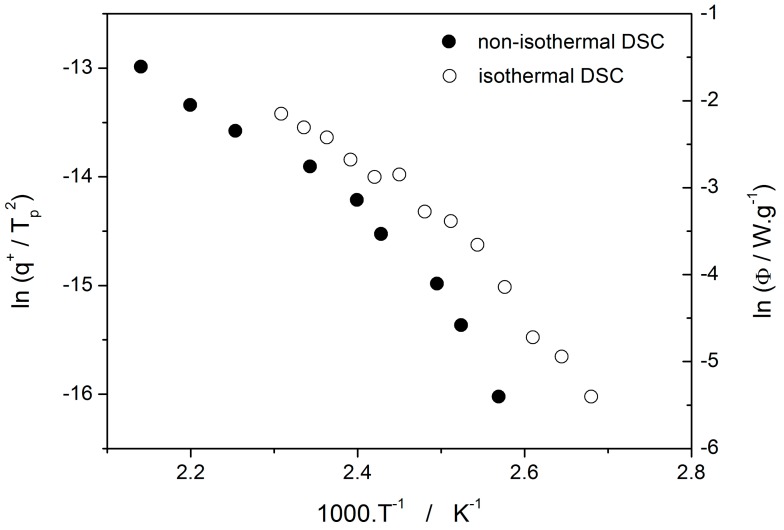
Determination of the crystallization activation energy for selenium glass by the Kissinger method (non-isothermal data) and the Friedman method (isothermal data).

The Kissinger and Friedman (for α = 0.50) plots are shown in Figure 7 for all the analyzed non-isothermal and isothermal DSC data, respectively. Similarly, as shown above for crystal growth experiments, also here it is seen that the activation energy significantly changes from relatively low values at a higher temperature (≅40 kJ·mol^−1^) to higher values at a lower temperature (≅117 kJ·mol^−1^).

Such important variation of the activation energy makes difficult further analysis of experimental data. The next step of kinetic analysis should be an estimation of the kinetic exponent *m* in Equation (10). Equation (9) can be rewritten in a somewhat different form,

(15) $$y\left(\alpha \right)=\phi \cdot \exp\left(E_{c}/RT\right)=\Delta H_{c}A\cdot f\left(\alpha \right).$$

**Figure 8:**
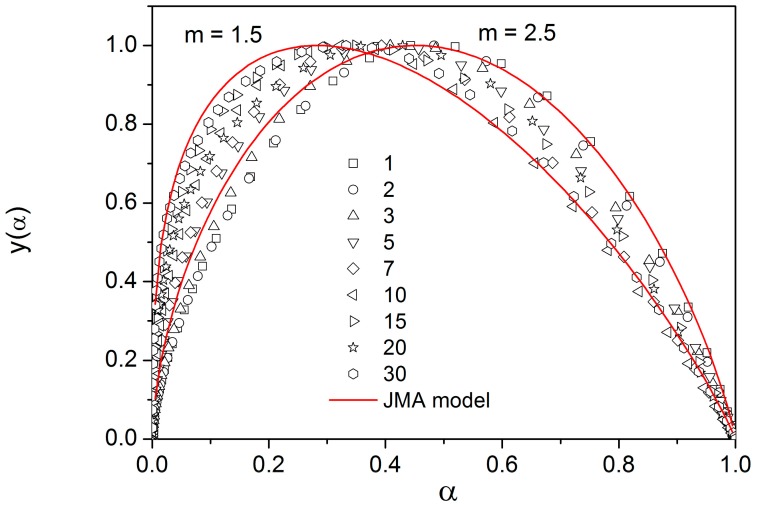
The y(α) function obtained by transformation of DSC data shown in Figure 5. Points were calculated by using Equation (15) (numbers indicate heating rate). Full lines were calculated by Equation (10).

Figure 8 shows the y(α) function obtained by transformation of all crystallization peaks from Figure 5 by Equation (15) for E_c_ = 105 kJ·mol^−1^. These plots are scaled within the 0 < y(α) ≤ 1 range for easier comparison of different data sets (points). Full lines shown in Figure 8 were calculated by Equation (10) for two different values of kinetic exponent (m = 1.5 and 2.5). It is clearly visible that a higher value of the kinetic exponent better fits data taken at lower heating rates, and lower values of m does the same for data taken at higher heating rates. However, this effect is artificial being just a consequence of important changes in activation energy discussed above.

**Figure 9:**
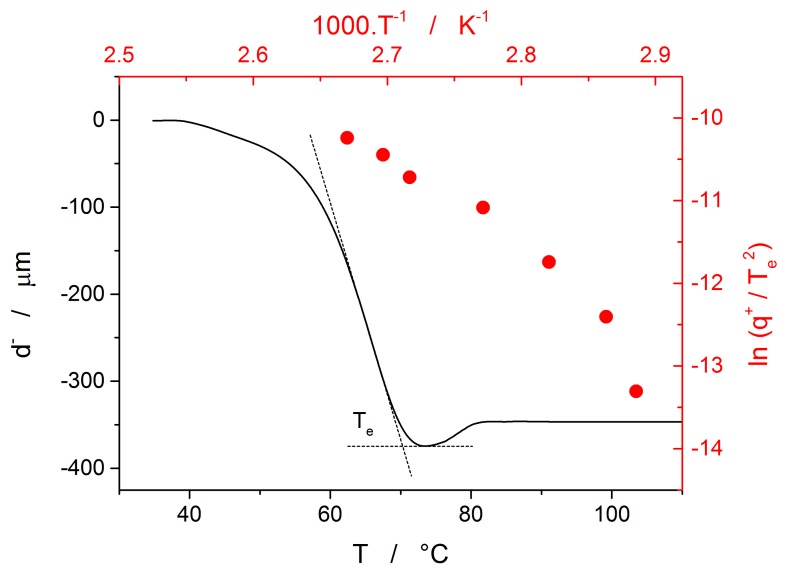
Black-based data and axes show an example thermomechanical analysis (TMA) crystallization curve (obtained at 0.2 °C·min^−1^); evaluation of the extrapolated endset is illustrated. Red-based data and axes show the Kissinger plot for the TMA crystallization data.

Crystallization in glassy selenium was also measured by means of thermomechanical analysis (TMA). The example curve obtained at 0.2 °C·min^−1^ is shown in Figure 9 as the temperature dependence of sample height decreased. As was shown in [22], one of most reliable and reproducible characteristic temperatures associated with the TMA crystallization measurements is the extrapolated endset temperature T_e_—its evaluation is suggested in the figure. The minimum achieved sample height is the point at which the sample deformation caused by viscous flow is ceased by the rigid crystalline structure formed within the sample. The occasional increase of sample height occurring during further heating is the consequence of the ongoing outwards surface crystal growth building up on the stiffened sample profile. The extrapolated endset temperatures can be utilized [23] in a similar way as the characteristic temperatures obtained via calorimetry, i.e., e.g., using the Kissinger equation (Equation (13)). The resulting dependence is for the glassy selenium depicted by the red data and axes in Figure 9. Note the curvature of the Kissinger plot data, similar to the one observed for the DSC crystallization data in Figure 7.

## 3. Discussion

Thermo-kinetic data described in the previous section imply that the activation energy of most kinetic processes observable in glassy materials by thermal analysis varies with temperature. This gives an interesting opportunity to compare the apparent activation energies among the particular processes for the wide range of experimental conditions. In such a situation, the equilibrium viscosity can be seen as the overarching quantity offering the most sensible comparison for all other data. The activation energy of viscous flow E_η_ can be obtained from the tangent slope for the viscosity data depicted in Figure 4:

(16) $$\frac{d\log\left(\eta \right)}{d\left(1/T\right)}=\frac{E_{\eta }}{2.303\cdot R}.$$

**Figure 10:**
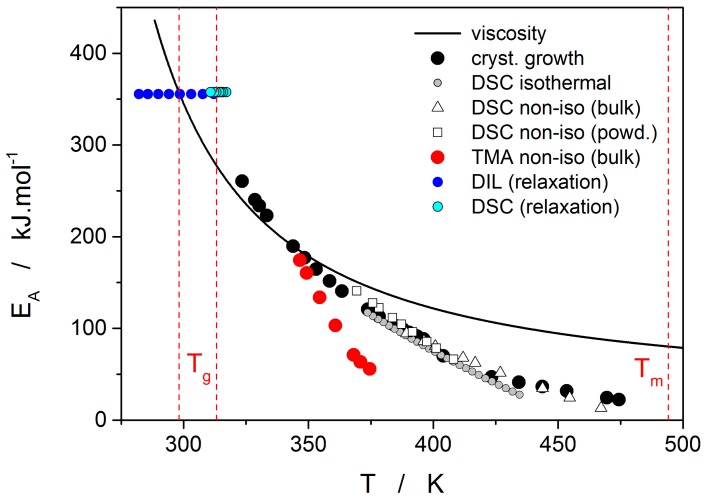
Activation energies of viscous flow (solid line), microscopic crystal growth, macroscopic crystallization observed by DSC (two data series, for bulk and powdered samples) and TMA, as well as structural relaxation observed by DSC and dilatometry.

The temperature dependence of E_η_ is shown in Figure 10 (solid line).

Starting with the structural relaxation process, it is somewhat surprising that the activation energy of this process remains constant throughout the whole measured temperature range (9–45 °C). Note that structural relaxation is generally considered to be very closely interlinked with viscous flow. Nonetheless, the constant value of E_rel_ in the above-given temperature range was unambiguously confirmed from the dilatometric data [3] (see, e.g., Figure 2), where E_rel_ was evaluated by non-linear optimization as well as by the linearization method [23], and also from the calorimetric data [3,24] (see, e.g., Figure 3), where the non-linear optimization was complemented by the newly developed methodology [25] based on the shift of the relaxation peak. Values of E_rel_ are shown in Figure 10 for dilatometry (dark blue) and calorimetry (light blue). It is well apparent that the relaxation data exhibit a constant value of E_rel_, not only in the glass transition temperature range commonly revealed via the non-isothermal measurement techniques (indicated by the red dashed lines), but also well below these temperatures. In the temperature window where the relaxation times considerably exceed the time-scale characteristic for the non-isothermal measurements, long-term isothermal annealing experiments need to be performed. Interestingly, the intersection of the E_rel_ and E_η_ well corresponds to the lowest value of T_g_ obtained during cooling within the commonly achievable time scale. The constancy of E_rel_ is a priori given by the definition of the TNM model, where the potential temperature-dependent component of E_rel_ is replaced by the T_f_-based term on the right-hand side of Equation (4). Direct incorporation of the E_rel_(T) dependence into Equation (4) might, however, be the way towards solving the occasionally raised questions [26] associated with the universality of TNM formalism—as such it is certainly worth of further exploration.

The second process occurring during further heating of the glassy materials is crystal growth, observable either microscopically (see the crystal growth data in Figure 4) or macroscopically, usually via calorimetric methods (see, e.g., Figure 5). Although the two approaches and the temperature ranges of their applicability differ to a great extent, the observed process is essentially the same and the corresponding activation energies should exhibit unified temperature dependence. In order to verify this hypothesis, E_G_ was determined from the crystal growth data depicted in Figure 4 by using Equation (7) (via the direct tangential approach), and E_c_ was determined from both non-isothermal and isothermal DSC measurements by using the Kissinger (Equation (13)) and Friedman (Equation (14)) methods, respectively (see Figure 7 for the two overall dependences). Again, the temperature-resolved tangential approach to the determination of E_c_ was adopted. The values of E_G_ and E_c_ are then compared in Figure 10, showing a very good agreement and confirming the universal nature of the activation energy for the crystal growth process. The non-isothermal DSC crystallization measurements were performed (in addition to the bulk samples) also for a finely powdered glassy selenium. Despite the different crystallization mechanism occurring in case of the fine Se powders [13,14], the corresponding E_c_(T) dependence also falls on the crystal growth master-curve depicted in Figure 10, further confirming the universality of this aspect of the crystallization process kinetics.

With regard to the relation between the crystal growth rate and viscosity, it results from the Turnbull–Cohen formula where u is inversely proportional to η. However, it has been shown [27] that for a number of materials the so-called decoupling of these two quantities occurs, breaking the Stokes–Einstein formalism [28]. Formally, the decoupling is described by the apparent decoupling parameter ξ_a_:

(17) $$\xi_{a}=\frac{d\log\left(u\right)}{d\log\left(\eta \right)}≅\frac{E_{G}}{E_{\eta }}.$$

This rather empirical expression can be further corrected by accounting only for the rate at which the structural entities (atoms, molecules) present in the liquid phase are attached to the growth liquid-crystal interface. This correction is based on the elimination of the term f_p_ from the expression for the crystal growth rate u, where f_p_ is the probability of the structural entity, newly attached to the crystal growth interface, remaining within the crystalline phase:

(18) $$\xi =\frac{d\left[\log\left(u\right)-f_{p}\right]}{d\log\left(\eta \right)}=\frac{d\left[\log\left(u\right)-\log\left(1-\exp\left(-\Delta G_{lc}/RT\right)\right)\right]}{d\log\left(\eta \right)}=\frac{d\log\left(u_{kin}\right)}{d\log\left(\eta \right)}≅\frac{E_{G,kin}}{E_{\eta }}.$$

ΔG_lc_ is the difference between the Gibbs energies of supercooled liquid and crystalline phases. Both forms of the decoupling parameter were essentially calculated from the ratio of the respective activation energies; ΔG_lc_ was calculated based on the standard thermodynamic expression [29] from the selenium enthalpy and entropy of fusion and the heat capacity data published in [30].

**Figure 11:**
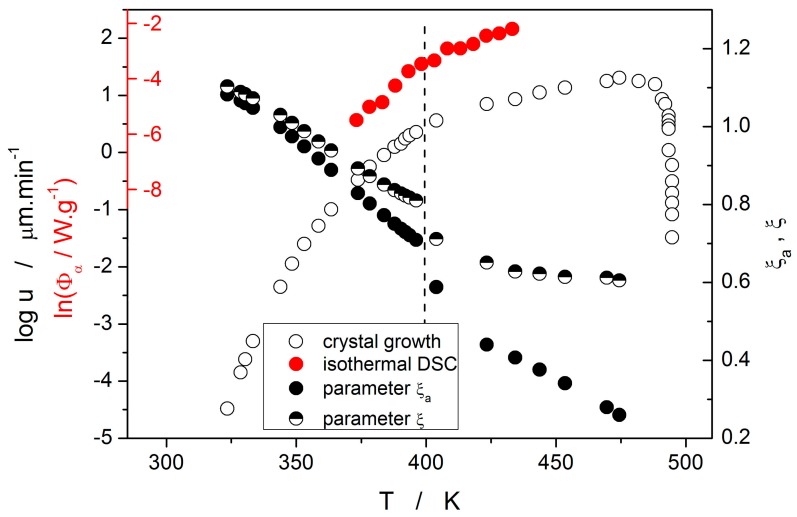
Temperature dependences of decoupling parameters ξ_a_ and ξ (right-hand axis), crystal growth rate u (black outer left-hand axis) and logarithm of heat flow corresponding to α = 0.50 obtained during isothermal DSC experiments (red inner left-hand axis). The vertical dashed line indicates the transition between the spherulitic B and A crystallite forms.

Temperature dependences of both forms of the decoupling parameter are shown in Figure 11. Values of ξ_a_ and ξ indicate that at low temperatures/growth rates almost no decoupling between u and η can be found (in fact, slight negative decoupling occurs below 340 K). However, as the temperature increases the decoupling becomes more prominent and very well recognizable at temperatures above 370 K. Increasing temperature also results in a rising difference between the two forms of decoupling parameters ξ and ξ_a_. This difference is negligible up to ~365 K but then rapidly increases with the exponential increase of the correction term f_p_. Note that the melting entropy of crystalline selenium is ΔS_m_ ≈ 1.5R [10]. Such a relatively small value in combination with low supercooling causes relatively large difference between ξ and ξ_a_ at higher temperatures. A similar effect may also bring N-type heat capacity of supercooled selenium [30], which effectively lowers the f_p_ contribution. Interestingly, both decoupling dependences exhibit a sudden step-like decrease of the decoupling parameter at 400 K. This is the consequence of the change in preferential morphology of the growing crystallites (transition from spherulitic form B to spherulitic form A, as described in [9]). The step-like change very well agrees with the break on the temperature dependences of the corresponding integral data utilized for calculation of E_G_ and E_c_, as depicted in Figure 11, where the dashed vertical line indicates the break point. It is noteworthy that the corrected rate of growth u_kin_ for the high-temperature spherulitic form A does not show further increase in decoupling (which would be represented by the decrease of ξ) and is close to ξ = 0.62.

The last data set depicted in Figure 10 is the one corresponding to the TMA crystallization measurements. Evaluation of activation energy from the TMA data was done similarly as in the case of non-isothermal DSC, i.e., by the tangential approach of the Kissinger dependence (Equation (13)), where the extrapolated endset T_e_ (see Figure 9) was used as the characteristic temperature. It was shown for several other chalcogenide glassy systems [31,32] that the activation energy evaluated in this way from the TMA measurements is in a good agreement with E_c_ from DSC, but the data are (due to the choice of the extrapolated characteristic temperature) shifted to lower temperatures; that is, the crystal growth process is seemingly observed “in advance” and the E_A_–T dependence gets shifted to lower temperatures. From this point of view, it is the high-temperature points shown in Figure 10 that in this dependence represent the commonly observed behavior. Contrary to what was observed for most other studied chalcogenides, E_A_ rapidly increases at lower temperatures. The exact position of T_e_ depends on many factors (including applied force, sample geometry, nucleation density, location and morphology of forming crystallites, etc.) but essentially can be understood as the competition between the viscous flow and crystal growth rate (which also depends on viscosity). In case of amorphous selenium, the position of T_e_ appears to be driven more by viscous flow (in combination with surface tension) at low temperatures. Thus, the activation energy determined from the TMA measurements at low q^+^ gets closer to E_η_.

**Figure 12:**
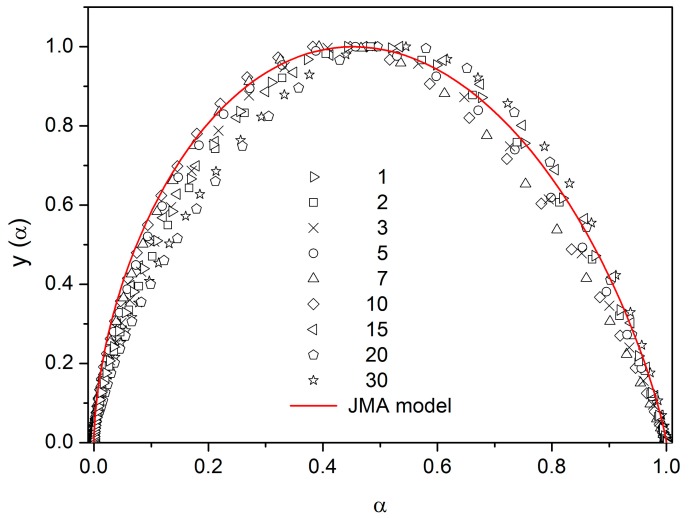
The y(α) functions obtained by transformation of DSC data shown in Figure 5. Points were calculated by using Equation (15) (numbers indicate heating rate); E_c_ utilized in the calculations were determined from the corresponding dependence shown in Figure 10 for the temperatures matching α = 0.1 of each respective DSC data curve. The solid red line was calculated by Equation (10) for m = 3.

There are of course many consequences associated with the temperature variation of crystallization activation energies. In the last part of the Discussion section we will focus on the model-based master-plot evaluation method utilizing the characteristic kinetic functions z(α) and y(α). The former can be expressed as the product f(α)·g(α), see Equation (11), and as such is invariable with E_A_. On the other hand, function y(α) is proportional to f(α), which utilizes E_A_ during the transformation of experimental data, see Equation (15). It naturally suggests itself to use the full E_c_(T) dependence (see Figure 10) in Equation (15). However, the y(α) function is too sensitive to the value of activation energy, and the large variation of E_c_ throughout each non-isothermal DSC measurement effectively results in y(α) distortions reminiscent of output obtained for the JMA formalism with the sub-unity kinetic exponent m. It is therefore reasonable to replace the full E_c_(T) dependence by constant values of E_c_, selected for each measurement individually based on the arbitrarily determined characteristic temperature point. If the value of E_c_ for the temperature corresponding to the maximum transformation rate (maximum of the DSC peak) is used, the resulting values of JMA kinetic exponents are too large to be physically meaningful. Nevertheless, if we consider that the dimensionality of the formed crystallites is being already set at the start of the crystallization process, and, correspondingly, we utilize E_c_ values corresponding to α = 0.10, the data depicted in Figure 12 are obtained.

As can be seen, most datapoints can be reasonably described by using the JMA kinetic exponent m = 3, which may correspond to the assumption that for bulk selenium samples the crystal growth starts dominantly via formation of three-dimensional volume-located crystallites.

## 4. Materials and Methods

Glassy selenium was prepared from pure elements (5N, Sigma-Aldrich, Prague, Czech Republic) by melt-quenching. Elementary Se was melted in an evacuated fused silica ampoule, which was then let to cool in air. The glassy material was crushed in agate mortar and sieved through defined mesh so that the various particle size fractions were obtained. Powder DSC crystallization data reported in this paper were obtained for the 20–50 µm fraction [13,14]. Pieces of glass with a diameter larger than 1 mm were used for the DSC relaxation and bulk crystallization measurements [3,13]. Melt-quench in thin ampoules was utilized to prepare cylindrical Se samples. The ampoules were quenched vertically to obtain a glassy ingot, which was then sawed into samples with the following diameters (d_m_) and heights (h_m_): For viscosity measurements d_m_ = 6 mm and h_m_ = 2.5 mm [12], for microscopic crystal growth measurements d_m_ = 4 mm and h_m_ = 2 mm [10] and for TMA crystallization measurements d_m_ = 4 mm and h_m_ = 1 mm.

Experimental setups and details of most measurements were already published in the respective papers: Structural relaxation by DSC and dilatometry in [3,24], viscosity in [12], microscopic crystal growth in [10] and non-isothermal powder and bulk crystallization by DSC in [13,14]. The new, previously unpublished data are those for isothermal bulk crystallization measured by DSC and non-isothermal crystallization measured by TMA.

The isothermal DSC data were obtained using a Q2000 DSC (TA Instruments, Prague, Czech Republic) equipped with a cooling accessory, autolid, autosampler and T-zero Technology. Dry nitrogen was used as the purge gas at a rate of 50 cm^3^·min^−1^. The calorimeter was calibrated using In, Zn and H_2_O. The stability of the DSC signal was checked daily. Open T-zero low-mass pans were used. Regarding the applied temperature program, the sample (8–10 mg) was first subjected to a 5 min isotherm at 45 °C and then heated at 100 °C·min^−1^ to a selected temperature *T_i_*, where the sample was allowed to isothermally crystallize until the crystallization process was complete. The isothermal crystallization temperatures utilized in the case of each particle size fraction were 100, 105, 110, 115, 120, 125, 130, 135, 140, 145, 150, 155 and 160 °C. In order to obtain a baseline for the isothermal measurement, each DSC pan with a crystalline sample of glass was kept in the DSC cell and the above-described temperature procedure was repeated (in this way the data subtracted from the isothermal crystallization signal truly surrogated the presence of an inert material with similar heat capacity, mass, grain size and positioning in the DSC pan/cell). Perfect flatness of the baseline and reproducibility of the crystallization measurements were confirmed. For the extensive testing of the suitability and repeatability of the initial 100 °C·min^−1^ heating ramp, see [15].

The thermomechanical measurements were realized by using a TMA Q400EM (TA Instruments), where the cylindrical samples were compressed in-between two alumina plates, and the force applied to the sample was 30 mN. A linear heating rate was applied to study the effect of crystal growth suppressing the decrease of sample height caused by viscous flow. The following heating rates were applied between 35 and 170 °C: 0.2, 0.5, 1, 2, 3, 4 and 5 °C·min^−1^.

## References

[1] Ediger M.D., Angell C.A., Nagel S.R. (1996). Supercooled liquids and glasses. J. Phys. Chem..

[2] Debenedetti P.G. (1996). Metastable Liquids. Concepts and Principles.

[3] Málek J., Svoboda R., Pustková P., Čičmanec P. (2009). Volume and enthalpy relaxation of a-Se in the glass transition region. J. Non Cryst. Solids.

[4] Naraynaswamy O.S. (1971). A Model of Structural Relaxation in Glass. J. Am. Ceram. Soc..

[5] Tool A.Q. (1946). Relation between inelastic deformability and thermal expansion of glass in its annealing range. J. Am. Ceram. Soc..

[6] DeBolt M.A., Easteal A.J., Macedo P.B., Moynihan C.T. (1976). Analysis of Structural Relaxation in Glass Using Rate Heating Data. J. Am. Ceram. Soc..

[7] Málek J., Svoboda R., Šesták J., Šimon P. (2013). Structural Relaxation and Viscosity Behavior in Supercooled Liquids at the Glass Transition. Thermal Analysis of Micro, Nano- and Non-Crystalline Materials.

[8] Málek J., Shánělová J., Šesták J., Šimon P. (2013). Crystallization Kinetics in Amorphous and Glassy Materials. Thermal Analysis of Micro, Nano- and Non-Crystalline Materials.

[9] Ryschenkow G., Faivre G. (1988). Bulk crystallization of liquid selenium—Primary nucleation, growth kinetics and modes of crystallization. J. Cryst. Growth.

[10] Málek J., Barták J., Shánělová J. (2016). Spherulitic Crystal Growth Velocity in Selenium Supercooled Liquid. Cryst. Growth Des..

[11] Bernatz K., Echeveria I., Simon S., Plazek D. (2002). Characterization of the molecular structure of amorphous selenium using recoverable creep compliance measurements. J. Non Cryst. Solids.

[12] Koštál P., Málek J. (2010). Viscosity of selenium melt. J. Non Cryst. Solids.

[13] Svoboda R., Málek J. (2013). Crystallization kinetics of a-Se, part 1: Interpretation of kinetic functions. J. Therm. Anal. Calorim..

[14] Svoboda R., Málek J. (2014). Crystallization kinetics of a-Se, part 2: Deconvolution of a complex process—The final answer. J. Therm. Anal. Calorim..

[15] Svoboda R., Málek J. (2015). Crystallization kinetics of a-Se, part 3: Isothermal data. J. Therm. Anal. Calorim..

[16] Svoboda R., Gutwirth J., Málek J. (2014). Crystallization kinetics of a-Se, part 4: Thin films. Philos. Mag..

[17] Málek J. (1992). The Kinetic Analysis of Non-Isothermal Data. Thermochim. Acta.

[18] Málek J. (1995). The applicability of Johnson-Mehl-Avrami model in the thermal analysis of the crystallization kinetics of glasses. Thermochim. Acta.

[19] Málek J., Criado J.M. (1990). The Shape of a Thermoanalytical Curve and Its Kinetic Information Content. Thermochim. Acta.

[20] Kissinger H.E. (1957). Reaction kinetics in differential thermal analysis. Anal. Chem..

[21] Friedman H.L. (1964). Kinetics of thermal degradation of char-forming plastics from thermogravimetry. Application to a phenolic plastic. J. Polym. Sci. Part C.

[22] Zmrhalová Z., Pilný P., Svoboda R., Shánělová J., Málek J. (2016). Thermal properties and viscous flow behavior of As_2_Se_3_ glass. J. Alloys Compd..

[23] Málek J. (1998). Rate-determining factors for structural relaxation in non-crystalline materials II. Normalized volume and enthalpy relaxation rate. Thermochim. Acta.

[24] Svoboda R., Pustková P., Málek J. (2007). Relaxation behavior of glassy selenium. J. Phys. Chem. Sol..

[25] Svoboda R. (2015). Novel equation to determine activation energy of enthalpy relaxation. J. Therm. Anal. Calorim..

[26] Kovacs A.J. (1963). Transition vitreuse dans les polymères amorphes. Etude phénoménologique. Fortschr. Hochpolym. Forsch..

[27] Ediger M.D., Harrowell P., Yu L. (2008). Crystal growth kinetics, exhibit a fragility-dependent decoupling from viscosity. J. Chem. Phys..

[28] Gutzow I.S., Schmelzer J.W.P. (2013). The Vitreous State: Thermodynamics, Structure, Rheology, and Crystallization.

[29] Busch R., Kim Y.J., Johnson W.L. (1995). Thermodynamics and kinetics of the undercooled liquid and the glass transition of the Zr_41.5_Ti_13.8_Cu_12.5_Ni_10.0_Be_22.5_ alloy. J. Appl. Phys..

[30] Svoboda R., Málek J. (2014). Thermal behavior in Se-Te chalcogenide system: Interplay of thermodynamics and kinetics. J. Chem. Phys..

[31] Svoboda R., Brandová D., Chromčíková M., Setnička M., Chovanec J., Černá A., Liška M., Málek J. (2017). Se-doped GeTe_4_ glasses for far-infrared optical fibers. J. Alloys Compd..

[32] Svoboda R., Brandová D., Chromčíková M., Liška M. (2019). Thermokinetic behavior of Ga-doped GeTe_4_ glasses. J. Non Cryst. Solids.

